# A New Experimental Porcine Model of Venous Thromboembolism

**DOI:** 10.3390/jcm10091862

**Published:** 2021-04-25

**Authors:** Leszek Gromadziński, Agnieszka Skowrońska, Piotr Holak, Michał Smoliński, Ewa Lepiarczyk, Anna Żurada, Mariusz Krzysztof Majewski, Mariusz Tomasz Skowroński, Marta Majewska

**Affiliations:** 1 Department of Cardiology and Internal Medicine, School of Medicine, Collegium Medicum, University of Warmia and Mazury in Olsztyn, Warszawska Str. 30, 10-082 Olsztyn, Poland; leszek.gromadzinski@uwm.edu.pl; 2 Department of Human Physiology and Pathophysiology, School of Medicine, Collegium Medicum, University of Warmia and Mazury in Olsztyn, Warszawska Str. 30, 10-082 Olsztyn, Poland; agnieszka.skowronska@uwm.edu.pl (A.S.); ewa.lepiarczyk@uwm.edu.pl (E.L.); mariuszm@uwm.edu.pl (M.K.M.); 3 Department of Surgery and Radiology with Clinic, Faculty of Veterinary Medicine, University of Warmia and Mazury in Olsztyn, Oczapowskiego Str. 14, 10-719 Olsztyn, Poland; piotr.holak@uwm.edu.pl; 4 University Clinical Hospital in Olsztyn, Clinic of Cardiology and Internal Diseases, Warszawska Str. 30, 10-082 Olsztyn, Poland; smolinskim@interia.eu; 5 Department of Radiology, School of Medicine, Collegium Medicum, University of Warmia and Mazury in Olsztyn, Warszawska Str. 30, 10-082 Olsztyn, Poland; anna.zurada@uwm.edu.pl; 6 Department of Basic and Preclinical Sciences, Institute for Veterinary Medicine, Nicolaus Copernicus University, Gagarina Str. 7, 87-100 Torun, Poland; skowron@umk.pl

**Keywords:** animal models of human disease, pulmonary embolism, venous thromboembolism, thrombosis

## Abstract

Venous thromboembolism (VTE), including deep vein thrombosis (DVT) and pulmonary embolism (PE), is a severe disease affecting the human venous system, accompanied by high morbidity and mortality rates. The aim of the study was to establish a new porcine VTE model based on the formation of the thrombus in vivo. The study was performed on 10 castrated male pigs: thrombus was formed in each closed femoral vein and then successfully released from the right femoral vein into the circulation of animals. In six pigs PE was confirmed via both computed tomography pulmonary angiography and an autopsy. Our research presents a novel experimental porcine model of VTE that involves inducing DVT and PE in the same animal in vivo, making it suitable for advanced clinical research and testing of future therapies.

## 1. Introduction

Venous thromboembolism (VTE) is a prevalent and life-threatening cardiovascular condition that requires accurate and timely diagnosis. It concomitantly includes deep vein thrombosis (DVT) and pulmonary embolism (PE) and usually is initiated by a thrombus formed in the venous system. Most of the pulmonary embolisms originate from DVTs of lower extremities, and approximately 50% of DVTs may lead to silent PE. Nearly 60% of patients with DVT develop PE as the thrombus travels to the lungs where it lodges in the pulmonary arteries [1,2]. VTE may be triggered by both the genetic factors, such as deficiency of natural anticoagulants [3,4], and acquired causes, including medical and behavioral reasons or drug therapy [5]. The most important and common risk factors associated with this disease are advanced age, surgery, cancer and immobilization [6].

Epidemiological studies have revealed that annually PE and DVT occur in 39 to 115 and 53 to 162 individuals per 100,000, respectively [1,7]. Mortality in PE patients is much higher than in those whose condition is limited only to DVT. The severity of VTE is best proven by the fact that every year, this disease is considered to be a major cause of approximately 500,000 deaths in Europe and over 300,000 deaths in the USA [8], and PE is the third most frequent reason of vascular death behind myocardial infarction and stroke [1,9].

Extensive investigations of new treatment strategies of VTE are still in progress. Animal models are used both to explain the mechanism leading to death in the course of this disease as well as to search for new therapies. Many different experimental models of PE involving rats, mice, pigs and rabbits have been described. The main problem is that the vast majority of these models are based on the formation of the thrombus ex vivo, i.e., outside the animal organism [10,11,12,13]. Hence, each new in vivo model of PE mimicking the “natural way” of thrombus formation, as well as its passage through the vascular bed, offers much more significant possibilities to investigate the pathophysiologic mechanism driving this disease. Therefore, the purpose of the present study was to develop a novel porcine VTE model based on the formation of the thrombus in vivo, which facilitates the simultaneous assessment of DVT and PE in one animal. We decided to employ the domestic pig in the experiment since it is considered to be one of the major animal species used in biomedical research. Furthermore, the porcine model has the coagulation cascade similar to that in humans, and the anatomy of the vascular network in pig hind limbs and human lower extremities is comparable [14,15,16].

## 2. Materials and Methods

### 2.1. Experimental Animals

The study was performed on 10 castrated male pigs (24 weeks old, 60 kg body weight, b.w.) of the Polish Landrace breed. The model of VTE was produced in 10 animals (the experimental group). All the animals were kept under standard laboratory conditions. They were fed standard fodder (Grower Plus, Wipasz, Wadąg, Poland) and had free access to water. The animals were housed and treated according to the guidelines of the local Ethics Committee for Animal Experimentation in Olsztyn (affiliated to the National Ethics Committee for Animal Experimentation, Polish Ministry of Science and Higher Education; decision No. 90/2018 of 13.02.2018). To ensure adequate acclimatization, the pigs were transported from the breeder to the animal quarters 5 days before the scheduled procedure.

### 2.2. Surgical Procedures in Experimental Animals

#### 2.2.1. Porcine VTE Model Development

Before performing any surgical procedures, all experimental pigs were pretreated with atropine (Atropinum Sulfuricum, Polfa, Warsaw, Poland, 0.05 mg/kg b.w., s.c.) and azaperone (Stresnil, Janssen Pharmaceutica, Beerse, Belgium, 2.5 mg/kg b.w., i.m.). Thirty minutes later, to induce anaesthesia, the main anesthetic drug propofol (Propofol-Lipuro, B. Braun Melsungen AG, Melsungen, Germany, 10 mg/kg b.w.) and the main analgesic drug ketamine (Bioketan, Vetoquinol, Poland, 10 mg/kg b.w.) were given intravenously in a slow, fractionated infusion. The depth of anesthesia was monitored by testing the corneal reflex. Once the animals were transported to an operating theater, general anesthesia during the entire procedure was maintained with inhalation of sevoflurane (Sevoflurane, Baxter, Ontario, CA, USA; administered at one human MAC end-tidal concentration 2.0%) under continuous pulse oximetry and heart rate monitoring. Under sterile conditions, the bilateral femoral veins were carefully exposed in each animal by dissecting the fascia and exposing and preparing the sartorius muscle. Next, both femoral veins were closed proximally and distally at the length of about 30–40 mm with surgical ligatures. To accelerate the thrombus formation, 200 units of thrombin (BioTrombina, Biomed Lublin S.A., Lublin, Poland) were administered into the closed segment of each vein. Immediately afterwards, to prevent the formation of thrombi outside the closed segments, a bolus of unfractionated heparin (Heparinum WZF, Warsaw, Poland) was administered, in a dose of 100 U/kg, through a catheter inserted in the ear vein. Two hours later (an optimal time needed for thrombus formation after closing of the femoral vein), the thrombus was released from the right femoral vein to induce PE. The thrombus release was facilitated mechanically by bending the right hind limb (thus the movements of muscles were acting as natural pistons). If this procedure was ineffective, 10 mL of saline was injected into the right femoral vein distally from the formed thrombus.

#### 2.2.2. Imaging Examinations Confirming PE

Computed tomography pulmonary angiography (CTPA; Aquilion PRIME TSX-303A Toshiba, Tustin, CA, USA), a method of choice serving to assess the pulmonary vessels when PE is suspected, was performed in all experimental pigs after the release of the thrombi from the right femoral veins. Using a maximum intensity projection (MIP) technique the thrombi were visualized and measured with the Toshiba Aquilion PRIME TSX-303A as standard software tool. During the CTPA procedure, the animals were sedated with propofol (Propofol-Lipuro, B. Braun Melsungen AG, 10 mg/kg b.w.).

#### 2.2.3. Euthanasia and Tissue Collection in Experimental Animals

Three hours after the CTPA, all experimental pigs were euthanized with sodium pentobarbital (Euthasol, FATRO, Ozzano dell’Emilia BO, Italy, 140 mg/kg). The entire heart and both lungs were dissected from each animal. After the incision of the right ventricle and the right atrium, the pulmonary trunk was incised immediately above the pulmonary valve. Then, very carefully and delicately, pulmonary arteries were successively cut open, beginning from the pulmonary trunk, and moving peripherally towards segmental and subsegmental arteries, to determine the size and location of the thrombus released from the right femoral vein. The left femoral vein was gently exposed and cut open to remove the thrombus with the tweezers.

## 3. Results

### Porcine Model of VTE

**Figure 1:**
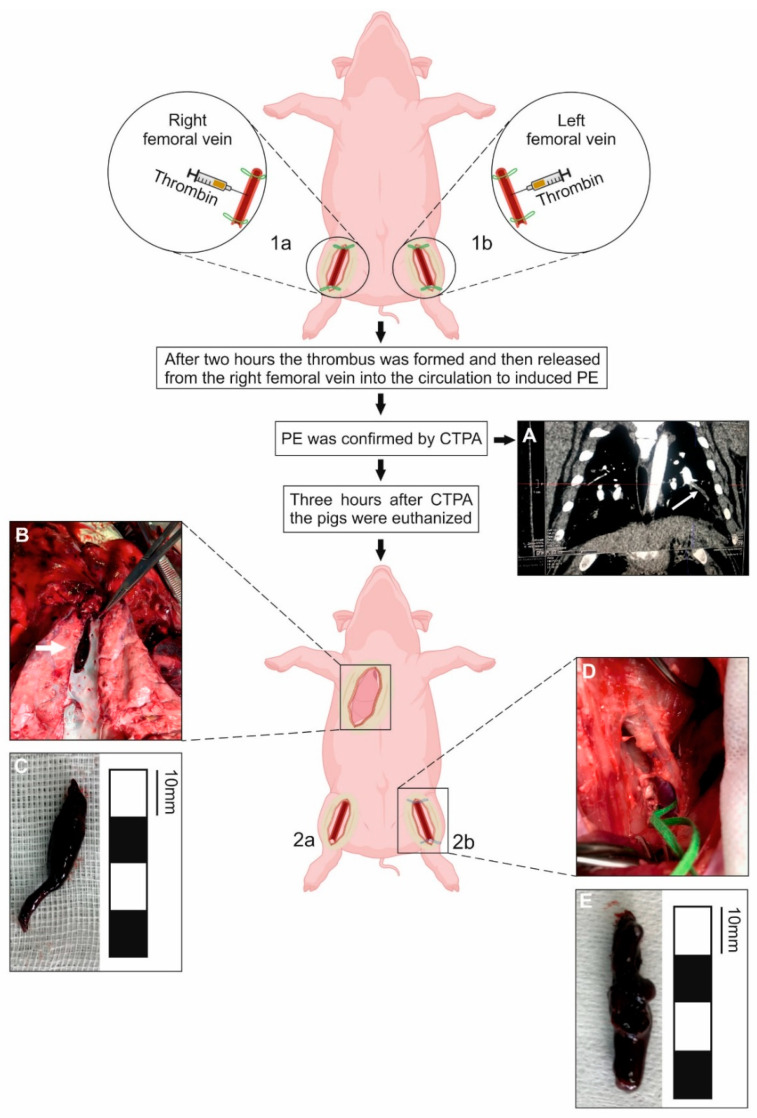
A porcine model of venous thromboembolism (VTE) based on the formation of the thrombus in vivo. The diagram represents the following stages of the porcine VTE model development. Both right (1a) and left (1b) femoral veins were closed proximally and distally at the length of about 30–40 mm with surgical ligatures. To accelerate the thrombus formation the thrombin was administered into the closed segment of each vein. Two hours later, the thrombus was released from the right femoral vein (2a) to induce pulmonary embolism (PE). PE was confirmed via both CTPA ((**A**) a representative CTPA scan; white arrow indicates the embolus in the pulmonary artery) and an autopsy ((**B**) a representative image of the embolus, indicated by a white arrow, in the left segmental pulmonary artery found at autopsy; (**C**) an embolus collected from the left segmental pulmonary artery). The left femoral vein with the remaining thrombus (2b) was gently exposed and cut open to remove the thrombus ((**D**) the exposed left femoral vein with the remaining thrombus closed with the surgical ligatures; (**E**) an image of the thrombus removed from the left femoral vein).

In all the experimental animals (*n* = 10) the thrombus was formed in each closed femoral vein and then was successfully released from the right femoral vein into the circulation. No significant changes in hemodynamic parameters were observed during the release of the femoral thrombus. In six of 10 experimental pigs, PE was confirmed via both CTPA, as well as an autopsy, finding the thrombi released from the right femoral veins in the segmental pulmonary arteries (Figure 1). In three pigs that did not develop PE, the released thrombi were found either in the right ventricle or in the right atrium (in one animal the formed thrombus was too large to pass through the pulmonary valve from the right ventricle to pulmonary arteries; in two pigs the thrombi were small, sized 5–8 mm, and were found within the system of the tricuspid valve). In one animal the released thrombus was neither found in the right heart nor in the pulmonary arteries (Table 1). Most likely, in this single case, the thrombus released from the right femoral vein underwent spontaneous fibrinolysis, or stuck in the venous system before reaching the heart.

## 4. Discussion

The present research provides an innovative in vivo porcine model of VTE. Since performing a detailed investigation of neither DVT nor PE on tissues obtained from humans proved to be impossible, several experimental animal models of these diseases have been used extensively. However, one of the major drawbacks of the currently available animal models of VTE is that most of them focus selectively only on one condition, either DVT or PE [9,17]. In respect to DVT, most investigations were performed in rodents [10,12] and all these models assume the application of factors enabling the creation of a thrombus in a vein. This can be achieved by the use of various chemical (ferric chloride solution) [18], photochemical (green light from a laser applied immediately before adding the diluted Rose Bengal, a photodynamic generator of oxygen free radicals) [19], mechanical (forceps) [20] or electrical (direct current which generates free radicals resulting in endothelial cell activation) [21] agents applied to the exposed vein, leading to thrombus formation by causing the transmural vessel injury. Another frequently used rodent model of DVT is a model of venous stasis, in which the formation of a thrombus is caused by complete and permanent occlusion of the investigated vessel, which reduces the venous flow and leads to both stasis-induced vein wall injury and enhanced tissue factor expression in endothelial cells and leukocytes [22,23,24]. In a rodent model of venous stenosis, the venous blood flow in the vessel is not stopped, but reduced by around 90%, which together with an activation of the endothelial factors at the stenosis site, leads to thrombus formation [25,26].

Although the use of the rodents in investigations of DVT has some advantages (such as relatively low cost and accessibility to genetically manipulated strains), rodents’ small body mass, short life span and high metabolic rate cause that they often respond to experimental interventions considerably different from humans. Thus, the need arose to introduce a new animal species in the investigations of DVT, which would allow for testing novel therapies and simultaneously would share more anatomical and physiological similarities with humans. Therefore, a porcine model has been proposed to investigate this disease since pigs share many similarities with humans, including the anatomical arrangement of blood vessels supplying the lower limbs and the coagulation cascade or even the cellular composition of the thrombus [27]. This was also the reason why we decided to introduce a new experimental model of VTE in this particular species. So far, three different models of DVT have been already proposed in pigs. The model described by Shi et al. [11] assumes the generation of DVT by inducing autologous thrombus into the inferior vena cava. In the stent-graft model of DVT, the development of a thrombus is achieved due to the venous stasis caused by installing a stent-graft in the common iliac artery [13]. The third model assumes the inducing of DVT by occlusion of the vessel, which can be either achieved by a balloon catheter [28], or by using surgical ligatures [29] (in both cases to facilitate thrombus formation, thrombin was injected into the isolated vein). Our experimental model of DVT bears a close resemblance to that proposed by Robinson et al. [29], as to induce the thrombus formation, the femoral veins were closed with surgical ligatures, and this process was accelerated with thrombin injection. However, as far as the previous works have only focused on DVT, our model goes one step further and enables the examination of the PE in the same animal.

So far, PE in animal models has been obtained with the use of several techniques [30]. The first one, applied in mouse, assumed intravenous injection of collagen and epinephrine into the right jugular vein which led to widespread platelet activation and subsequent (lethal) PE [31]. In the mouse model described by Matsuno et al. [32], PE was evoked by continuous induction of thrombus in the left jugular vein via endothelial injury, due to a photochemical reaction. Nonetheless, in the majority of animal models of PE, the thrombus causing this disease was formed outside the animal organism and then injected into the vessel [33,34,35]. In the present experiment, we succeeded in introducing the porcine experimental model in which not only both DVT and PE are induced in the same animal but also both these conditions are initiated in vivo. Only few such attempts have been made so far. Rectenwald et al. [36] succeeded in creating PE in rats by formation of endogenous thrombus (or placement of an inert silicone “thrombus”) in the inferior vena cava and releasing it two days later to embolize to the lungs. In dogs, DVT was triggered in an isolated segment of the internal jugular vein by inducing local phlebitis with exposure to sodium morrhuate, and then the venous patency was re-established to induce PE [37]. As far as now, one attempt has been made to induce both DVT and PE in pigs in which a thrombus was generated using intravascular thrombin and collagen injection between occlusive balloons placed in the inferior vena cava; 90 min later these balloons were deflated and removed to release the thrombus [37,38]. However, in contrast to the previous experiments, we aimed at recreating the natural course of PE in terms of formation of the thrombus in femoral veins (as in people the natural course of VTE frequently begins with formation of the thrombus in the lower limbs vessels) and then releasing it to embolize in the lungs. Consequently, we definitely believe that the novel animal model proposed by our research group is one of the clinically most relevant for studying pathomechanisms and advanced therapies of VTE.

## 5. Potential Limitations

Like all experimental models, ours has some limitations that must be recognized. Firstly, some clear interspecies differences in coagulation profile exist between animals and humans. However, it should be emphasized that recent data confirms the potential usefulness of pigs as an experimental species to study human diseases involving initiation of the clotting cascade [39]. Secondly, the creation of thrombus in our experimental model requires the application of external factors, thus, it is difficult to entirely relate DVT pathophysiology in pig to man. However, it must be stressed that this problem refers to all VTE or DVT animal models. Moreover, in the present study the histopathological examinations of the thrombi were not performed. For this very reason, in the nearest future, we plan to use our model to investigate the possible differences in the composition of the thrombi from each left femoral vein and the thrombi that embolize in the lungs. Additionally, although the biggest advantage of our novel porcine model is inducing VTE in vivo, it must be mentioned that this goal was not achieved in all the experimental animals, as in 4 from 10 experimental pigs, the thrombus released from the vein did not develop PE. In the present experiment however, the thrombus was released from only one (right) femoral vein, and in our opinion in some animals the amount of it could be too small to embolize in the lungs. The confirmation of this thesis may be finding small sized thrombi within the system of the tricuspid valve. We believe that a possible solution to this problem is releasing the thrombi from both (left and right) femoral veins. We plan to test this hypothesis soon, but in all likelihood this modification could result in much higher efficiency of the created animal model of PE.

## 6. Conclusions

In conclusion, the current research presents a novel experimental porcine model of VTE that involves inducing DVT and PE in the same animal in vivo. Such an innovative approach allows to analyze a natural course of the pathomechanisms underlying the development of PE and may be valuable in studies concerning reactions between the thrombus and the femoral vein or pulmonary artery endothelium. Therefore, we hope that our research will lay the groundwork for future clinical trials and new therapeutic agents.

## Figures and Tables

**Table 1:** The characteristics of the location and size of thrombi found in autopsy and computed tomography pulmonary angiography (CTPA) imaging.

Experimental Pig Number	The Location and Size (mm) of the Thrombi Found in Autopsy	The Location and Size (mm) of the Thrombi Measured in CTPA Images
1	lack of the thrombus	lack of PE
2	LPA 30 mm	LPA 25 mm
3	LPA 35 mm	LPA 40 mm
4	RPA 31 mm	RPA 21 mm
5	RV 180 mm	lack of PE
6	RV-TV 5 mm	lack of PE
7	LPA 40 mm	LPA 25 mm
8	RV-TV 8 mm	lack of PE
9	LPA 35 mm	LPA 30 mm
10	LPA 30 mm	LPA 35 mm

PE—pulmonary embolism, LPA—left pulmonary artery, RPA—right pulmonary artery, RV—right ventricle, TV—tricuspid valve, CTPA—computed tomography pulmonary angiography.
